# Toward a relational biodiversity economics: Embedding plural values for sustainability transformation

**DOI:** 10.1073/pnas.2314586122

**Published:** 2025-09-26

**Authors:** Jasper O. Kenter, Rachel Carmenta, Michael Christie, Hywel Griffiths, Eberechukwu Ihemezie, Adrian Martin, M. Teresa Gomez-Osorio, Unai Pascual, Christopher M. Raymond, Kyriaki Remoundou, Ruth Waters

**Affiliations:** ^a^Aberystwyth Business School, Aberystwyth University, Aberystwyth SY23 3DY, Wales, United Kingdom; ^b^Ecologos Research Ltd, Aberystwyth SY24 5LR, Wales, United Kingdom; ^c^Department of Environment and Geography, University of York, York YO10 5DD, United Kingdom; ^d^School of Global Development, University of East Anglia, Norwich NR4 7TJ, United Kingdom; ^e^Tyndall Centre for Climate Change Research, University of East Anglia, Norwich NR4 7TJ, United Kingdom; ^f^Department of Geography and Earth Sciences, Aberystwyth University, Wales SY23 3DB, United Kingdom; ^g^Basque Centre for Climate Change, Scientific Campus of the University of the Basque Country, Leioa 48940, Basque Country, Spain; ^h^Ikerbasque Basque Foundation for Science, Bilbao 48009, Basque Country, Spain; ^i^Helsinki Institute of Sustainability Science and Ecosystems and Environment Research Program, Faculty of Biological and Environmental Sciences, University of Helsinki, Helsinki 00014, Finland; ^j^Department of Economics and Management, Faculty of Agriculture and Forestry, University of Helsinki, Helsinki 00014, Finland; ^k^Natural England, Worcester WR5 2NP, United Kingdom

**Keywords:** IPBES, relationality, biodiversity economics, environmental values, pluralistic valuation

## Abstract

The prioritization of market over nonmarket values of nature is a key driver of the global biodiversity crisis. Recognizing nature’s diverse values in decisions is a fundamental lever for sustainability transformation. While economic valuation of nature has a long history, it has struggled to recognize the full suite of nature’s values, particularly the broad, relational, intrinsic, and shared values reflecting the complexity of human–nature relationships. We explore opportunities to expand the consideration of values within the economics of biodiversity by reviewing conventional and heterodox economic approaches. We argue that integrating pluralistic values requires a relational biodiversity economics that transcends people–nature dualism and seeks the flourishing of life. We synthesize foundations for such a paradigm in relation to worldviews, values, value indicators, and life frames. Our perspective transcends the dominant economic framing of nature as a passive, largely substitutable asset, to also consider nature as place, self, and harboring agency. This helps to overcome the limitations of conventional economic assumptions, better reflects peoples’ lived experiences, and supports transformations toward more just and sustainable futures.

Unsustainable human activities are driving global biodiversity loss, with severe consequences for human well-being (1). Complex, interconnected social, institutional, and economic factors drive ongoing degradation, including economic inequality, corporate vested interests, and exclusion of marginalized communities in decision-making (2, 3). A key driver of this decline is that economic systems prioritize market values and short-term material wealth, displacing nonmarket values (4, 5). This is reflected in dominant economic indicators like Gross Domestic Product (GDP). Major environmental economic reports, such as The Economics of Ecosystems and Biodiversity (TEEB) (6) and the Dasgupta Review (7) advocate nonmarket approaches to value nature’s benefits to people and integrating these values into economic decisions through market (e.g., fees, taxes, subsidies) and nonmarket instruments (e.g., standards and quotas). This enables more efficient allocation of biodiversity benefits across society and generations. However, as these influential reports recognize, valuation of such ecosystem services has almost exclusively considered nature’s instrumental values (i.e., nature as a means to human well-being) and the aggregation of individual preferences, overlooking other types of values that could further strengthen the case for biodiversity conservation (4).

The Values Assessment by the Intergovernmental Platform on Biodiversity and Ecosystem Services (IPBES) (8) strongly calls for the recognition of more diverse values in decisions, beyond individual, instrumental values, echoed by the Kunming-Montreal Global Biodiversity Framework (9). This broader set of values includes *relational* values (values of meaningful, nonsubstitutable relationships with, and enabled through nature); *intrinsic* values (values for nature independent of human welfare); *broad* values (overarching principles and life goals); and *shared* values (collective values that people form through social processes, as distinct from aggregated individual values) (10–12). The Values Assessment highlights that integrating a broader range of nature’s values into decision-making supports more sustainable, just, and inclusive decisions, enhancing multidimensional well-being, and designing interventions that enable and sustain proenvironmental individual and collective behaviors. Conversely, failing to recognize diverse values can undermine public acceptance of decisions, marginalize people who prioritize noninstrumental values, and exacerbate social and environmental conflicts and harms, especially for vulnerable and historically marginalized groups (8).

Economists studying biodiversity increasingly recognize diverse value concepts. The TEEB framework acknowledges the sociocultural constituents of well-being that fall outside of conventional economic indicators (6), and the Dasgupta Review recognizes the salience of nature’s intrinsic values, the foundational importance of nature to human health and well-being, and worldviews that consider nature’s agency and personhood (7). However, progress toward integrating and applying such concepts in economic studies of biodiversity has been limited (4, 13). The reasons for this are well established: Environmental economists conventionally view nature as a passive resource separate from people, values as individualistic utilitarian preferences measured via willingness to pay (WTP), and social value as the aggregation of individual preferences (14). People are assumed to be self-interested, rationally maximizing their own well-being. Where regard for others exists, this is assumed as embedded in self-regarding preferences; altruism is not considered to exist outside of contributing to individual utility (10).

Relational, intrinsic, and shared values are difficult to align with these assumptions. Yet, such values influence people’s decisions regarding their interactions with nature at multiple scales: for example, people spend time and money on nature conservation charities and acts of stewardship without clear instrumental benefits to themselves, and decision-makers also allocate resources toward nature protection and institute rights of other-than-human entities that reflect their intrinsic values, including through legislation. Relational values, connecting nature and people via notions like identity, place, heritage, care, stewardship, and personhood, play important roles in how nature is managed and how rights and obligations over it are allocated (15, 16), and such decisions are often based on shared rather than individual values (17, 18). However, because this diversity of values is not typically institutionalized in economic decision-making procedures, metrics, and tools, many of nature’s values frequently end up being overlooked, or in conflict with conventional economic assessments (8).

IPBES highlights the need for new ways of thinking about economics that can account for noninstrumental and nonindividual values, recognizing that they are incommensurable within conventional economic framing because they are difficult (or inappropriate) to incorporate into preference-based trade-off analyses (e.g., cost–benefit analysis) or monetize (8). It also argues that new approaches are needed to support institutional shifts away from the broad values of individualism and materialism emphasized by conventional economic models to more community-based sustainability-aligned values (5), and to recognize the diverse worldviews that can support such shifts, including those of Indigenous peoples and local communities (4). These calls reflect the broader economic scholarship of heterodox, “new” economic approaches like ecological, feminist, well-being, doughnut, degrowth, and postgrowth economics that advocate transforming economics toward a focus on holistic human and planetary well-being (19, 20). “New” in this context does not refer to approaches being recent, but to the need for economics as a discipline to transform and establish a new mainstream (21).

However, a synthetic perspective on how to incorporate more diverse values in biodiversity economics remains absent. To address this, we sketch the foundations for a relational perspective that embeds value pluralism, reviewing conventional and new economic approaches to values and developing a novel theoretical synthesis, structuring insights using the IPBES typology of nature’s values. In this context, relationality means considering people, nature, and their values as coconstituted by their relations with each other, as complex assemblages of living processes (22, 23), with relationships fundamental to people and nature’s beingness and identity, and to human and planetary well-being.

We go beyond previous attempts at addressing these issues in economics (e.g., 14, 24, 25) by considering them not just at the level of values, but also in relation to worldviews and ontologies of human–nature relationships (26, 27). Our argument reflects a broader “relational turn” in the humanities, social, and sustainability sciences, which seeks to better capture the complexity of human–nature relations by understanding them as dynamic and continually unfolding, and emphasizes embodiment and lived experience rather than mental representations of nature external to people (28). This paradigm shift supports more holistic social–ecological systems thinking, greater epistemic justice for Indigenous and local peoples, and transforming governance toward more sustainable sets of shared values and ethics of care (5, 28, 29). Crucially, it offers a more accurate characterization of what it means to be human than the atomistic rational-actor model of conventional economics, as ignoring people’s relationality inevitably leads to misunderstanding human values and behavior and fails to reflect people’s lived experience of nature and themselves. This can lead to apparently economically efficient decisions nonetheless provoking substantial social conflict (8, 10).

In developing a relational biodiversity economics, we first examine conceptualizations of nature’s values by conventional environmental economics and IPBES. Next, we explore opportunities to integrate diverse values through the expansion of established environmental economic frameworks and new economic approaches, concepts, and methods. Building on new economic perspectives, we establish the conceptual foundations for a relational economics of biodiversity that embraces plural values through relational ontologies, to underpin more participatory, holistic, and inclusive valuation and appraisal approaches. Finally, we discuss how a relational turn in biodiversity economics can strengthen responses to the deepening global biodiversity crisis and enable just sustainability transformations.

## Conceptualizing Nature’s Diverse Values

We broadly conceive biodiversity economics as the area of economics that studies the role of nature, biodiversity and ecosystems in the production, consumption, valuation, allocation, and exchange of goods and services, how those economic processes affect nature, and the way they are institutionalized and governed (c.f. 19). It mostly falls within the broader conventional field of environmental economics but is also studied by heterodox approaches, particularly ecological economics. Biodiversity economics has for decades recognized that many of nature’s values are poorly expressed through markets and, therefore, often ill-considered in decisions. The conceptual framework of Total Economic Value (TEV) arose as a central effort to address this (6), organizing nature’s values into direct use values (e.g., using timber for building materials), indirect use values (e.g., the way ecosystems regulate floods), option values (the values placed on maintaining options for future benefits), and nonuse values (the value placed on maintaining nature for other people, nonhuman species, and future generations). In addition, the (natural) insurance value of nature conservation reflects the benefits of protecting ecosystems to offer resilience against adverse events like natural hazards and mitigates the costs of risks to wider ecosystem services (30). Such values tend to be measured in monetary terms, which is appealing to policymakers as it allows values to be directly compared using a single value indicator. To achieve this, individual values are aggregated to social scales for use in decision-support tools like cost–benefit analysis and natural capital accounting (6) and macroeconomic indicators like Inclusive Wealth (7).

**Fig. 1.**
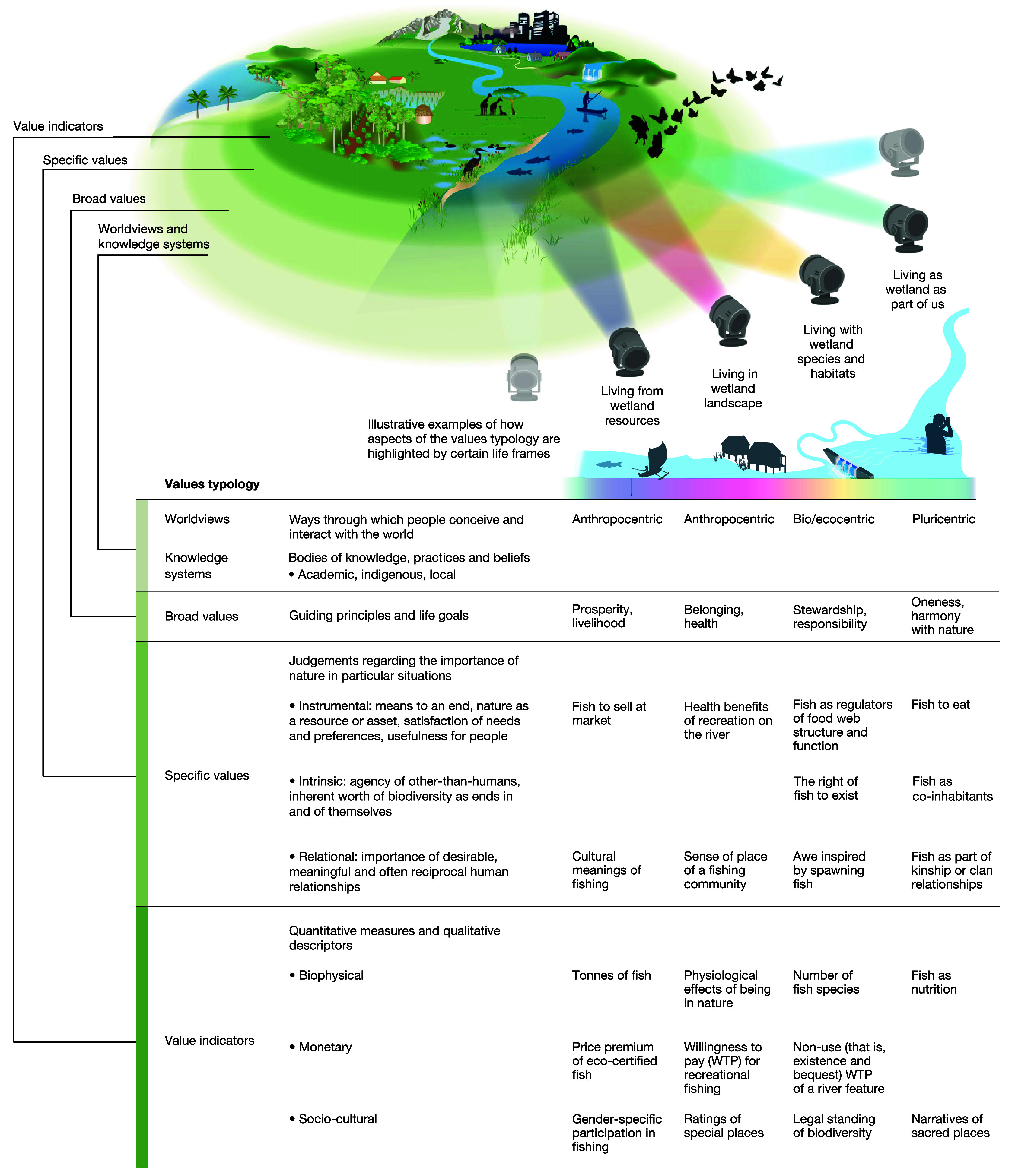
The IPBES Values Typology. Concentric circles illustrate different layers of value (worldviews, broad and specific values, value indicators). The four *life frames* depict how the different ways people frame their relation to nature implies prioritizing certain values across these dimensions. Life frames are not mutually exclusive; individuals or groups can express multiple frames. Examples illustrate values that might be highlighted in the context of a freshwater ecosystem. Reprinted from ref. 4, which is licensed under CC BY 4.0.

The IPBES Values Assessment points out that TEV is primarily oriented toward instrumental values grounded in an anthropocentric and ontologically dualistic (i.e., nature as separate from humans) worldview, which constrains transformation toward just and sustainable futures (8). To address these limitations, the Values Assessment offers a more pluralistic and inclusive typology of nature’s values (12). It draws on a wide range of scientific disciplines and perspectives to create a multilayered framework that incorporates different worldviews and knowledge systems, broad values, specific values, and value indicators. These are organized across four *life frames* of human–nature relationships, where people live *from*, *with*, *in*, and *as* nature, as a heuristic to articulate different lenses through which values may be understood (4, 31) (Fig. 1). This challenges the predominance of *dualism* that is prevalent in the economics of biodiversity and opens up space for more holistic and relational ontologies of human–nature relationships (27).

The *worldviews* and *knowledge systems* layer highlights that people with different backgrounds, experiences, and geographies may interact with and view biodiversity from many different epistemic and cultural perspectives. This challenges the predominance of *anthropocentrism* in the economics of biodiversity and creates space for biocentric and ecocentric worldviews, where nature is inherently worthy of respect, and pluricentric worldviews, where people and nature are seen as reciprocal and interdependent (32). Relational worldviews span a spectrum from pluricentric to “weak” anthropocentric worldviews, where there is a recognition of the human dependence upon relationships with nature, including for culture (8, 33). There are also biospheric and ecocentric relational worldviews, including in political, deep, and social ecology (22). Epistemically, better integration of more diverse scientific, Indigenous, and local, knowledge systems challenges *reductionism*, allowing for greater reflection of the richness, complexity, and dynamism of economies, values, and people–nature relationships (34).

*Broad values* (e.g., wealth, fairness, belonging, harmony with nature) are life goals and guiding principles that transcend particular contexts. They are informed by worldviews, institutionalized and expressed through social norms and legal rules, and orient specific values and individual and collective environmental behaviors (12). *Specific values* are the values that people express in relation to a particular context. Specific values for nature include *instrumental*, *relational*, and *intrinsic* values. In contrast to instrumental values, intrinsic and relational values are nonsubstitutable (11), e.g., the intrinsic and relational values of a woodland that reflect the inherent worth of its biodiversity and sociocultural connections to people cannot be simply replaced or compensated for by another woodland. Specific values may be expressed through monetary, biophysical, and sociocultural *value indicators*.

Finally, broad and specific values are also frequently expressed and formed collectively as *shared values*, through long-term communication and socialization processes and shorter-term group deliberations. Shared values challenge ontological and methodological *individualism*, including assumptions that values can be isolated from their social–ecological context and are fundamentally individual, and that benefits and costs to society can be simply aggregated from the individual to social scale (10). For example, things that are pursued for individual (often instrumental) benefits can lead to societal harms that undermine shared and relational values, such as where income-generating activities (e.g., agricultural modernization) erode social cohesion, ecosystem services, and shared livelihoods (35).

Altogether, the IPBES typology challenges assumptions of *value monism, instrumentalism, anthropocentrism*, and value *substitutability* and *commensurability*, where values are considered through a single ontological, epistemic, and ethical framework, and nature is solely considered important as a substitutable means to satisfying human preferences, which can be appropriately measured, aggregated, and traded-off in monetary terms. While these challenges draw on long-standing critiques (14), they are nonetheless still poorly addressed by biodiversity economics, without a cohesive pluralistic framework for economic valuation emerging. In the next section, we will review diverse opportunities and approaches for integrating plural values into biodiversity economics with particular attention to approaches that align with the relational turn in sustainability sciences.

## Opportunities and Approaches for Integrating Plural Values in Biodiversity Economics

There are multiple pathways for better integration of plural values within biodiversity economics, including pragmatically adapting established frameworks and drawing on approaches that more fundamentally challenge conventional assumptions. Below, we first consider opportunities within TEV as an established environmental economic framework and the use of Inclusive Wealth as a key indicator proposed by the Dasgupta Review to help operationalize nature’s values at the macroscale (7). We then consider how insights from behavioral economics extend our understanding of value motivations in economic decisions. Third, we explore how diverse, heterodox “new” economic (19) approaches with more relational conceptions of nature, people, and well-being could support a more complete adoption of the IPBES values typology.

While TEV is theoretically grounded in individual, instrumental preferences, empirical evidence suggests that TEV can be shaped by broader values. In practice, empirical studies eliciting the nonuse components of TEV have revealed diverse motivations underlying stated preferences, including rights, duties, care, and stewardship pertaining to biodiversity, which align with a variety of broad, relational, intrinsic, and shared values (18, 36, 37). While the relationship between the constituents of TEV, monetary indicators like WTP, and noninstrumental values have been the subject of long-standing conceptual debate (38–40), further empirical investigation could clarify the relationships between TEV-in-practice and diverse value concepts, opening up a space for more informed discussion about when and how it is feasible and appropriate to monetize such values (41). For example, TEV assumes that nature’s values are, at least in principle, substitutable by other means to satisfy preferences. Relational values are often considered nonsubstitutable, because they are context dependent and typically place-based and nontradable (42). Yet, some empirical research suggests that substitutability of relational values may differ with place-attachment and across income groups (43). There may also be differences in substitutability depending on whether values are considered in terms of gains or losses. Thus, in some contexts, TEV-based valuation could be appropriate to assess noninstrumental values to some extent. Beyond this, biodiversity economists have suggested instruments such as regulatory limits to impose quantity constraints where nonsubstitutable intrinsic and relational values are beyond the scope of TEV (7).

At the macrolevel, Inclusive Wealth has been promoted as a superior indicator of economic performance compared to GDP. It provides a coherent framework to monitor societal progress toward sustainability through accounting for the value of all capital assets, including natural capital. Where accurate market prices do not exist, nature’s values are considered through “shadow prices,” which represent aggregate marginal contributions to welfare via the TEV framework. However, measuring the value of natural capital assets using monetary indicators can be empirically challenging, and existing measures of Inclusive Wealth currently only measure a subset of nonmarket values (44), which risks incomplete assessments of progress toward sustainability. Improving the way economics maps nature’s diverse values to TEV could enable a more accurate understanding of the degree to which these values can be reflected in Inclusive Wealth. There is thus potential for better understanding and expanding the breadth of values reflected by TEV, Inclusive Wealth, and similar indicators. However, this potential is constrained by instrumental framings of nature as capital assets providing flows of ecosystem services to people and the predominantly reductive, anthropocentric worldview underpinning it.

Behavioral economics has focused on various challenges to conventional economic assumptions of people as rational self-interested utility maximizing agents, recognizing diverse noninstrumental motivations. Behavioral economics considers people as influenced by their relationships and their social and institutional context. Experimental evidence highlights that social norms, other-regarding preferences, and broad values like fairness, trust, and reciprocity affect economic behavior (45, 46). Behavioral economists have developed new theories and models of choice and behavior to account for the influence of these broader motivations. For example, dual motive theory highlights how preferences are underpinned by distinct self- and other-regarding motivational pathways (47), and nudge theory highlights how it is possible to change behaviors through changing an individual’s choice architecture (48). These perspectives can help design policy instruments and interventions. Green nudges can encourage proenvironmental behaviors (48), while recognizing social norms, culture, and trust is important for the successful implementation of payments for ecosystem services schemes to incentivize nature conservation (49). There are also opportunities for better integration of fairness and social norms in economic valuation, for example, in studies that elicit “fair prices” through deliberative valuation (50). Behavioral economics thus meaningfully expands the analysis of relations between people and nature to include diverse social and noninstrumental motivations. While it has not explored relationality at the level of worldviews or challenged dualism between people and nature, its more nuanced models of human behavior and the implications of its empirical findings can provide bridges for dialogue between conventional and more relational heterodox perspectives.

“New” economics represents diverse areas of heterodox scholarship and practice that further challenge conventional assumptions of instrumentalism and individualism. While new economics has no consensus definition, it comprises diverse normative perspectives that emphasize holistic views of human and planetary well-being, ecological limits, and equity, and embrace the transformation of social and economic systems, values, beliefs, worldviews, and paradigms needed for long-term sustainability of the biosphere (19, 20). Recent synthesis (19) has identified a set of principles common to many new economic approaches, including relationality, social–ecological holism, complexity, embeddedness, participation, equity, and multidimensional understandings of well-being. Such principles reflect foundations in more relational worldviews that shape broad and specific values. For example, institutional economics considers economic relationships as social relationships, which means they need to be considered within a social and institutional context (51). It thus follows that values as social constructs are formed, and privileged or marginalized, through institutional means (e.g., norms, rules, rights) (52). Ecological economics considers economic relationships as social–ecological relationships, including through the notion of the throughput of materials and energy as “societal metabolism” (53) and understanding ecological sustainability as the relation between the “scale” of economies and the ecosystems they are embedded within (54). It also sees justice and the environment as closely related, understanding justice as a good, sustainable, dignified life for both humans and other beings (55).

Various indigenous-held societal perspectives like *Buen Vivir*, *Ubuntu*, and *Kaitiakitanga* express diverse new economic principles (19) and consider social–ecological relationships in their own terms, emphasizing broad values such as reciprocity and respect (56, 57). The worldviews that underpin them are grounded in more holistic understandings of human–nature relationships, with people coconstituted by their social and ecological contexts (32, 56). For example, Ubuntu fosters a relational ethics that can support ecological justice and fairness, respecting rights, fellowship and reconciliation, strengthening communal bonds and harmonious relationships, and supporting care and mutuality (58, 59). This underpins many opportunities for inclusive economic decision-making in Africa, e.g., in equitable resource allocation and natural resource conflict resolution (60).

Feminist, care, and some areas of development economics have focused on value in terms of the satisfaction of needs and enabling capabilities of people to achieve the lives they value (61–63), rather than just fulfilling individual and material preferences. Here, needs (including relationships with nature) are considered largely nonsubstitutable in their fulfillment and socially embedded in their contribution to well-being, which is itself considered a multidimensional, relational construct. Relational well-being can be understood as arising from common life and shared enterprise within communities, with relationships considered both the means through which needs are met and through which material and nonmaterial (e.g., psychological, symbolic) goods are distributed (64). Feminist economics points out that “women’s work” (work historically associated with women, within and outside of markets) lies at the heart of this. Women’s work and nature are both foundational to economies, and historically undervalued and exploited (65, 66). There are also important parallels between their relational values, being typically place-based, socially and ecologically embedded, embodied, and associated with ethics of care (65, 67). Care ethics emphasizes the importance of empathy and emotion in moral judgment and action. It understands the self as constituted by relations to other beings, with a focus on actual, context-specific, unique relationships, challenging reductive thinking (67).

Nature’s values in new economic approaches are frequently considered as shared (i.e., socially generated and intersubjective), with both instrumental and relational dimensions, sometimes coupled with a recognition for nature’s intrinsic values, and diverse notions of respect for other species. Broad values like dignity and sufficiency are often considered as more important than unconstrained preference satisfaction, and essential for achieving sustainability transformation (5, 68). This is reflected in relational concepts like *homo integralis* and fields of practice like humanistic management that reflect people’s wholeness, dignity, and social–ecological embeddedness, and provide an alternative to models of human beings as rational utility maximizers (19, 69, 70).

Moving away from individualism and instrumentalism conceptually opens the way for methodological approaches that are more cognizant of the relational and shared dimensions of nature’s values and do not assume value substitutability and commensurability. For example, there is a long tradition of applying participatory methods in the Global South (e.g., participatory action research, participatory rural appraisal) that focus on the lived experience of communities and people in relationship, rather than as atomistic agents. An important lesson from these approaches is that they do not just provide a means to an end (e.g., identifying desirable actions and policies) but can also themselves enhance justice, generate social learning, and directly benefit the well-being of participants (71, 72).

In ecological economics, participatory approaches have been primarily implemented through deliberative valuation methods. Deliberation has been utilized to form shared values and bridge conflicting values, presenting an alternative to resolving value trade-offs through optimization models. Questions on how to allocate environmental goods or public resources can be informed by groups directly establishing the social value of different options through, e.g., deliberative monetary valuation or social multicriteria approaches that can combine monetary and nonmonetary indicators (73–75). In these cases, numerical estimates of value can be agreed upon for policy options through deliberation without assuming commensurability of underlying values. For example, in a deliberative monetary valuation of a marine plan in Scotland that incorporated various options to protect biodiversity, a representative group of citizens was asked to assess the relative value of the plan as a percentage of local taxes, while comparing that value to other social expenditure (e.g., education, social care, police) as a benchmark (75). By deliberatively valuing potential policy options for protecting or managing biodiversity at the social scale, any number of considerations considered relevant by citizens could be reflected in the valuation without commodifying environmental goods and their relational and intrinsic values. While such methodological approaches have thus far been limited in their scale of application, they have substantial potential to improve the positive policy impact of biodiversity valuation studies (8). There is also potential to integrate them with more established policy tools like participatory budgeting, which has seen large-scale application, particularly in South America. Participatory budgeting involves communities deliberating on decisions on how to allocate public funds, which can lead to more accountable and greener spending decisions (71).

A further area of biodiversity economics with significant potential for integration of multiple values through increased participation relates to Payments for Ecosystem Services (PES) and other conservation incentive programs. PES are often designed in alignment with promarket visions of the governance of nature, commanding multibillion-dollar annual investments worldwide (76). PES conventionally constitute incentives for securing the flow of valuable ecosystem services and in so doing protecting nature as a capital stock. Narrow instrumental conceptualizations, however, have largely ignored the wider range of values meaningful to both the beneficiaries and stewards of nature’s services, contributing to gender inequality, power asymmetries, and epistemic bias toward Global North perspectives (77). There is increasing evidence that both more participatory and more relationally framed approaches positively impact effectiveness (e.g., through social legitimacy and equity) of PES programs on the ground, while reducing perverse outcomes such as crowding out intrinsic motivations for conservation (78–80). Relationally framed PES approaches could align well with weak anthropocentric relational worldviews that balance peoples’ interdependence with nature with its instrumental benefits.

At the macroeconomic level, new economic approaches like ecological, well-being, degrowth, and postgrowth economics have emphasized the importance of holistic economic progress indicators, where various aspects of nature’s values are integrated, as alternatives to GDP (19). These metrics have been adopted by an increasing number of countries. This may involve composite measures, such as Inclusive Wealth, or dashboard approaches like Cymru Wales’ Well-being of Future Generations Indicators (81) and Aotearoa New Zealand’s Living Standards Framework (82). The latter provides one of the most advanced examples of institutionalizing nature’s multiple values in economic policy evaluation. It considers well-being at three levels: 1) individual and collective multidimensional well-being (e.g., health, knowledge, housing, belonging, environmental amenity, subjective well-being); 2) the role of political, economic, social, and cultural institutions (e.g., families and households, civil society, firms, and markets) in facilitating well-being; and 3) the wealth of Aotearoa New Zealand, including aspects of wealth related to nature, human capabilities, and social cohesion that are not fully captured in the system of national accounts. The framework integrates many relational aspects of Māori knowledge systems, worldviews, and values with western relational concepts of well-being and value (83) and can be leveraged to prioritize nature–well-being relationships in policy (84). It has recently removed natural capital concepts from its framework and replaced them with a more holistic framing that emphasizes noninstrumental ontologies of nature (82) and anchors plural values in relational worldviews and a pluralistic understandings of human–nature relations. The framework is hosted by Aotearoa New Zealand’s Treasury Department, is considered core rather than supplemental to economic policy assessment, and has a formal role in policy evaluation, priority setting, and public resource allocation. It shows that it is possible to integrate nature’s values in economic decision-making without assuming the commensurability and substitutability of different values through a single metric (contrasting it with, e.g., Inclusive Wealth), and without disregarding or trying to monetize relational and intrinsic values of nature, addressing long-standing concerns of commodification associated with environmental valuation.

## Toward a Relational Biodiversity Economics

We have discussed the need to integrate nature’s multiple values in biodiversity economics, and examples of conceptual shifts, methods, and approaches in research and policy demonstrating how this can be achieved. What becomes evident is that moving beyond conventional economic assumptions does not just challenge individualistic, monistic, and instrumental conceptions of nature’s values in economic analysis, but also underlying anthropocentric worldviews and dualistic ontologies of nature. Building on relational thinking in new economics and the relational turn in sustainability science more broadly (22, 28), we set out a relational approach to biodiversity economics that cuts across the layers of the IPBES values typology, considering its characterization and implications in terms of life frames, worldviews and knowledge systems, broad values, specific values, and value indicators (Table 1).

**Table 1.** Implications of a relational biodiversity economics in terms of the different aspects of nature’s values associated with the IPBES values typology

Value aspect	Conventional biodiversity economics	Relational biodiversity economics
Life frames (framing of people–nature relations)	Nature is primarily considered through a living *from* nature frame. Nature is framed as a passive and largely substitutable resource to be used optimally: a stock of natural capital providing a flow of ecosystem services.	Human–nature relations are considered more pluralistically, according to multiple life frames. Nature is recognized as a resource (*living from nature*) but equally considered in terms of life processes (*living with nature*), place (*living in nature*), and self (*living as nature*).
Worldviews	Anthropocentric worldviews, with humans implicitly at the apex of a hierarchy, and a dualistic ontology of nature where nature is considered as separate from people and culture.	Relational worldviews where people are coconstituted by their relations with nature and each other, moving beyond people–nature dualism. Relational worldviews include a spectrum between relational (weak) anthropocentrism and relational biocentric, ecocentric, and pluricentric worldviews. Economic relationships are seen as socially, institutionally, historically, and ecologically embedded.
Knowledge systems	Emphasis on quantitative, technocratic knowledge; economics works in parallel with other knowledge systems in studying environmental issues. Optimal solutions for allocating resources primarily seen as a technical matter.	Deeper integration between economics and other scientific and local and indigenous knowledge systems, and between quantitative and qualitative approaches, with an emphasis on participation in research and policy and understanding lived experience. Optimal solutions for allocating resources primarily seen as a matter of social choice.
Broad values	Limited consideration. Utility-maximization and efficiency are implicit normative goals. Different broad values are considered commensurable and can be traded-off against each other through preference utilitarianism and the concept of individual utility. Well-being (welfare) is considered as individual preference satisfaction; broad values pertaining to nature are deemed to be reflected in individual preferences.	Explicitly considered. Broad values conceived of pluralistically and can be expressed in terms of diverse western and nonwestern ethical systems. Care for life is a common normative orientation. Well-being is conceived of as holistic and multidimensional, and the quality of relationships between people and between people and nature considered as key aspects. Human and planetary well-being considered interdependent.
Specific values	Primary focus on instrumental values, expressed as individual, self-regarding preferences. Intrinsic and relational values considered indirectly as nonuse values insofar as they can be termed as instrumental preferences. Intrinsic and relational values also recognized through quantity restrictions. Individual values considered as preformed; values of individuals not considered interdependent. Social values considered the aggregate of individual values. Other-regarding preferences acknowledged in behavioral economics.	Considers instrumental, intrinsic, and relational values. Values considered as more than preferences including through concepts like capabilities, needs, and rights. Intrinsic and instrumental values of nature considered coemergent with relational values. Values recognized as expressed within a relational context and intersubjective; values of individuals considered interdependent. Social values considered as shared values arising from social processes.
Value indicators	Emphasis on coupling monetary and biophysical indicators. Sociocultural indicators of nature’s values generally considered the domain of other disciplines. Specific values assumed commensurable through monetary metrics. Indicators mostly deemed generalizable and transferable between contexts. Methodological individualism and focus on optimization models to inform economic resource allocation.	Integration between biophysical, monetary, and sociocultural indicators. Emphasis on dashboard and multicriteria approaches, deliberation, and participatory appraisals as ways to bridge multiple indicators and provide more holistic evaluation and metrics to guide economic resource allocation. Indicators often more context dependent. Shift away from optimization to determining appropriateness of actions salient to specific people and their relationships to specific places.

In terms of the IPBES life frames, a relational economics of biodiversity recognizes nature as more than a bundle of resources (living *from* nature) or other species and ecological functions worthy of conservation (living *with*), also considering nature as place (living *in*), and as self, harboring its own agency (living *as*). These understandings reflect diverse, relational worldviews where there is no clear division between nature and people, and life processes are not conceived of as belonging to either nature or culture (85). People and nature are entangled, and physical, biological, social, cultural, and economic processes emerge from relationships (22), as do values (23). Economic relationships are thus seen as ecologically, socially, and institutionally embedded. This limits the usefulness of reductionistic epistemic approaches that isolate people or the natural environment from the relational sphere.

In terms of broad values, a relational biodiversity economics explicitly recognizes diverse values beyond the conventional normative economic goals of prosperity and efficiency, recognizing a plurality of ethical systems and building on economic traditions that focus on capabilities and needs. A central normative orientation is care for life (human and nonhuman) (67). In terms of specific values, relationality does not imply the exclusion of intrinsic and instrumental values; all broad and specific values can be considered through the lens of relational worldviews (32). From this perspective, instrumental values signify the means-end aspects of ecological economic relationships (23). While intrinsic values have been conceived both within and beyond the realm of human valuation and preferences (42), regardless of their precise conceptualization, their articulation in practice arises within a relational field, from where people may express intrinsic, relational, and instrumental values in a layered or intertwined way (27). The notion of values arising from a relational field also enables a relational biodiversity economics to move beyond individualism and more prominently consider shared values (both broad and specific) formed intersubjectively through diverse social processes.

Embedding a relational approach in biodiversity economics is a formidable challenge, requiring transdisciplinary conversations around value indicators and methods that can bridge diverse, often incommensurable values and indicators, and integration between economics and other disciplines to not just elicit instrumental and noninstrumental values in parallel, but link sociocultural, biophysical, and monetary valuation to underpin holistic resource allocation. The integration of relational worldviews and pluralistic life frames in economic analysis and institutions for economic decision-making is essential. This means going beyond adapting existing conceptual frameworks like TEV or indicators like Inclusive Wealth to better recognize relational values. Integration at the levels of specific values and indicators alone risks exacerbating instrumentalism and commodification of nature by falling into the trap of suggesting that all values can ultimately be translated and expressed in preference utilitarian terms (86). In turn, this could ultimately reinforce dominant broad values around individualism, instrumentalism, materialism, and profit-maximization that form substantial barriers to just sustainability transformation (4, 5). Sophisticated dashboard approaches like Aotearoa New Zealand’s Living Standards Framework and deliberative valuation and participatory appraisal methods are therefore important, as they provide mechanisms for identifying priorities without assuming that different values are commensurable. However, there are also trade-offs between meaning and scale, and between complexity and resourcing of valuation and appraisal methods. Well-established, relatively resource-efficient desk- and survey-based TEV approaches and methods like cost–benefit analysis do not need to be abandoned altogether but need to be more explicit in terms of assumptions and limitations, while more elaborate pluralistic and participatory decision-support tools and policy instruments are appropriate for more complex and contested decision-making contexts.

Bringing about a shift to a relational biodiversity economics both involves and supports transformative societal change (i.e., fundamental, system-wide change). This requires shifts in foundational beliefs, values and worldviews, and the institutions and power structures that these imaginaries are inscribed into, and the myriad operational decisions that are in turn shaped by them. For a relational turn in biodiversity economics to become transformative thus requires going beyond more and better valuation of the environment to also embrace fundamental shifts toward worldviews and knowledge systems that can accommodate more diverse values and, by extension, the formalization and legitimization of such values in institutions and economic systems (87), challenging power structures and vested interests associated with strongly anthropocentric, dualistic worldviews as well as human inequalities.

The Values Assessment is explicit in arguing that taking more relational perspectives is essential for sustainability transformation. This is a pluralist argument, meaning that the call for better recognition of relational values and worldviews does not imply that other types of values are inherently “bad,” but that degradation of nature is linked to the domination of instrumental and individualistic values at the cost of relational, intrinsic, and shared values. There is now considerable agreement with the idea that relational perspectives are aligned with sustainability goals, not only in the specific literature on relational values but also in bodies of literature on sustainability transitions and transformations and in futures scenarios (5). For example, the Values Assessment reviewed 460 sustainability scenarios, finding that those scenarios associated with better futures for people and nature assume people express nonmaterial broad and shared values like solidarity, care, and justice, which are more actively considered by relational perspectives, while those associated with declines in nature and human well-being tend to assume continued individualism and materialism (8).

Adopting more relational models and approaches that recognize diverse values is thus essential for expanding the values lens within the biodiversity field, and “updates” biodiversity economics with the advent of relationality within the broader discipline, including diverse new economic approaches. It also aligns with research that indicates that people who recognize diverse values in land management, often underpinned by relational worldviews, can achieve more effective biodiversity conservation (18, 88, 89). Recognition of more diverse values and relational worldviews can also increase participation in environmental land use schemes (90), increase justice in environmental conflicts (91), and may be associated with greater life satisfaction (92). Relational and intersubjective aspects of values play important roles in realizing diverse capabilities and well-being benefits through engagement with nature, such as emotional connection, spiritual realization, and perception, imagination, and other cognitive and sensory functions, and enhance empathy and regard for others and nature, potentially leading to more altruistic behaviors in resource use (93–95). In relation to broad values, given that these are thought to change more slowly than specific values, there are substantial opportunities to identify sets of existing latent values (e.g., around responsibility, justice, and inclusion) as meaningful leverage points for transformation (92). Regarding valuation, there is a limited evidence base of monetary valuation studies that explicitly implement a relational approach, such as via deliberative prompts that aim to bring out relational perspectives, or by integrating methods like storytelling or place-based walking discussions. This evidence shows a widening of the scope of values considered, discernable social learning, and shifts toward more holistic thinking and greater care, though not necessarily higher willingness to pay (73, 75, 96). Given the more established benefits for justice and sustainability of broader participatory approaches (32, 71, 72), validating and better understanding such effects is an important area of future research.

## Conclusion

Economics shapes everyday decision-making from the local to the global scale. A relational turn in biodiversity economics offers new transformative pathways toward just and sustainable futures, drawing on more holistic concepts, methods, and metrics to better account for social and environmental impacts, transform economic institutions, and enable genuine, inclusive, sustainable economic progress. Such transformations are essential to deliver the UN-CBD Ecosystem Approach and Kunming-Montreal Global Biodiversity Framework, which emphasize the importance of community participation and integration of multiple values, worldviews, and knowledge systems (9, 97).

Transforming biodiversity economics to more fully recognize relational worldviews and diverse values can help bridge the gap between the wide range of values people express and those taken up in resource allocation decisions. A relational biodiversity economics supported by effective deliberative and participatory methods offers powerful pathways for making economic decisions more inclusive, addressing value conflicts more explicitly, and increasing the transparency and impact of economic evidence (96, 98). This can strengthen democratic support for the major and urgent societal shifts required to address the global biodiversity crisis and wider polycrisis. Recognizing the embeddedness of people and economies in nature also enables the increased consideration of nature’s broader role in society, such as in health and education. By more holistically considering human motivations and the context-specific meaning of relationships, relational perspectives can reorient biodiversity economics toward the lived experience of people and their interactions with nature.

Key avenues for advancing relational biodiversity economics include systematically identifying best practices on how relational worldviews and value concepts can be embedded across the IPBES values typology through different methods, tools, and approaches across research and policy; in what ways these practices generate different outcomes for biodiversity, sustainability, justice, and social-economic benefits like human capabilities and social cohesion; and how to overcome barriers and vested interests toward their adoption and institutionalization.

## Data Availability

There are no data underlying this work.
